# A Case of Thermal Esophageal Injury Induced by Sodium Picosulfate with Magnesium Citrate

**DOI:** 10.1155/2017/9879843

**Published:** 2017-06-04

**Authors:** Dong-Hyuk Yang, Byoung Wook Bang, Kye Sook Kwon, Hyung Kil Kim, Yong Woon Shin

**Affiliations:** Division of Gastroenterology, Department of Internal Medicine, Inha University School of Medicine, Incheon, Republic of Korea

## Abstract

Although thermal esophageal injuries caused by hot food or tea have been reported, thermal esophageal injury due to sodium picosulfate with magnesium citrate (PSMC) used for bowel preparation is rarely reported. We report the case of a 56-year-old man who presented with esophageal injury after ingestion of PSMC. Instead of dissolving the PSMC in water before ingestion, he drank water immediately after swallowing PSMC powder. As soon as he drank water, he developed severe chest pain and hematemesis. Upper endoscopy revealed severe hemorrhagic, ulcerative mucosal change from upper to mid-esophagus. He was hospitalized for nine days, received conservative treatment (fasting and parenteral nutrition), and recovered without complications. When PSMC is used as a colonic cleansing agent, patients should be educated to take it after dissolving it sufficiently in 150 mL of water to avoid esophageal thermal injury.

## 1. Introduction

Colonoscopic examination is increasing in Korea as a screen for colorectal cancer [1]. Bowel cleansing agents are very important for successful colonoscopy. Polyethylene glycol and PSMC are widely used bowel cleansers. Polyethylene glycol is the most commonly used laxative. However, it may cause adverse effects such as nausea, vomiting, and abdominal discomfort. In addition, it may be rejected due to its unpleasant odor and taste [2]. PSMC, which has a similar bowel cleansing effect, is used for easier administration. PSMC is a dual-action laxative that has an orange scent. The active components include sodium picosulfate, magnesium oxide, and citric acid. When PSMC is dissolved in water, magnesium oxide and citric acid combine to form magnesium citrate, which acts as osmotic laxative [2], and sodium picosulfate, a stimulant laxative [3]. Due to its dual action, PSMC is a powerful laxative and patients comply well because of the pleasant fragrance. However, inappropriate use of PSMC can cause serious side effects [2, 3]. When it contacts water, an exothermic reaction occurs; therefore it should be dissolved in water and cooled before taking it. Here, we report a case of thermal injury of esophagus with acute bleeding caused by incorrect use of the PSMC.

## 2. Case Report

**Figure 1:**
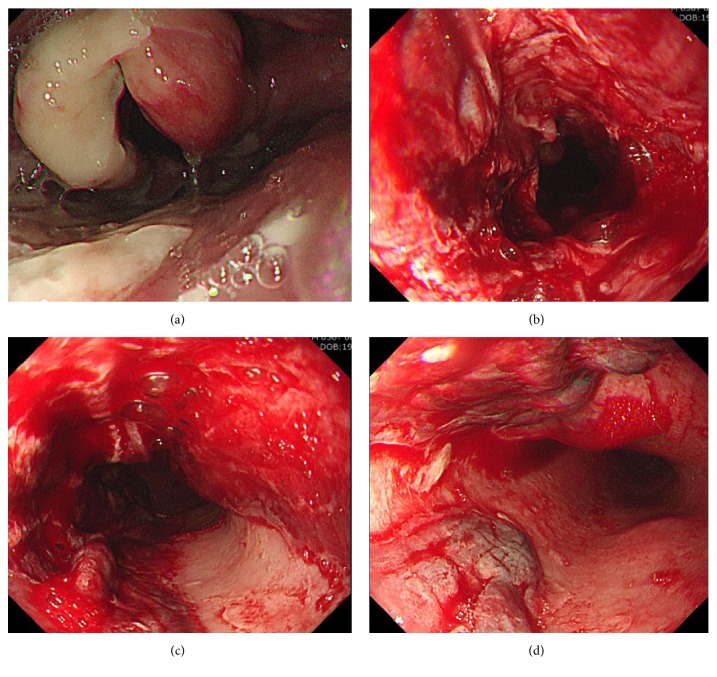
Upper endoscopy at admission. The epiglottis appeared swollen with moderate mucosal injury (a). Diffuse edematous and friable mucosa with deep ulceration and hemorrhage from the upper (b) to mid-esophagus ((c) and (d)).

**Figure 2:**
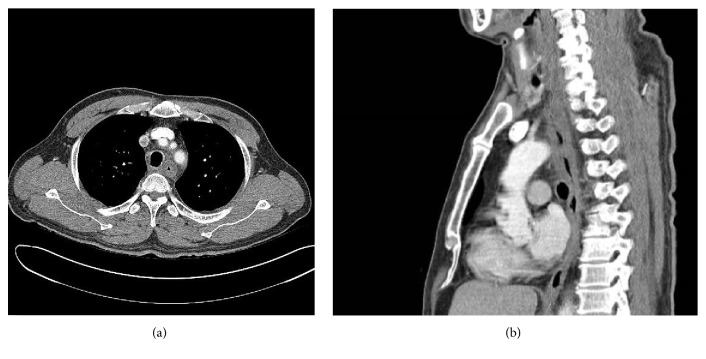
Chest computed tomography (CT). Transverse plain (a) and sagittal plain (b) images of the chest CT showed diffuse esophageal wall edema and thickening. However, esophageal perforation or mediastinitis was not detected.

**Figure 3:**
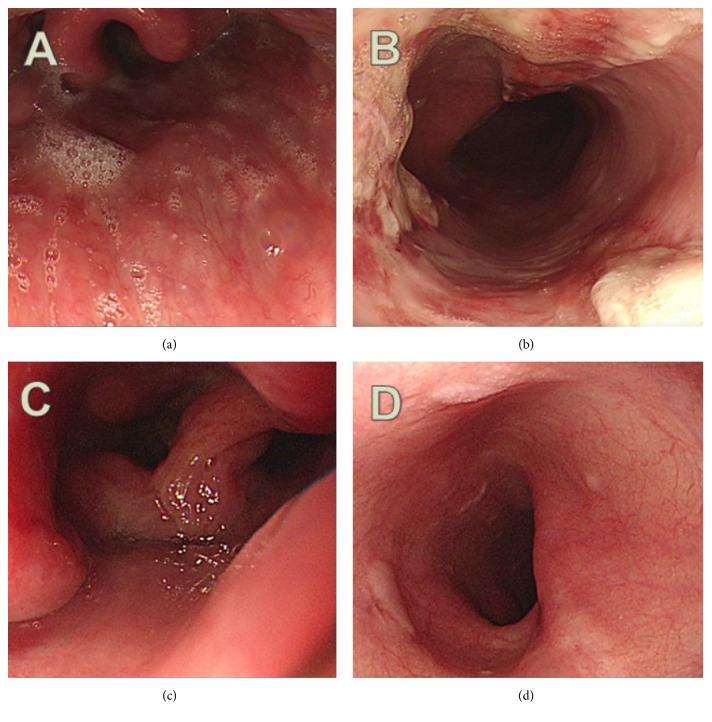
Upper endoscopy after conservative treatment. Endoscopic finding one week after thermal injury ((a) and (b)). The epiglottis was nearly healed and diffuse ulcerative lesions of the esophagus were improving without more bleeding. Endoscopic finding one month after thermal injury ((c) and (d)). The epiglottis was completely healed and whitish scars were seen in the esophagus.

A 56-year-old man visited our hospital with severe chest pain and hematemesis. He was scheduled to receive colonoscopy in a local hospital. He did not sufficiently dissolve the PSMC powder in 150 mL of water as described in the drug instructions. Instead, he swallowed the PSMC powder and immediately drank water. Instantly after drinking water, he felt severe chest pain and shortly thereafter vomited about a cup of blood. On arrival at the emergency room, he was hemodynamically stable and alert. His blood pressure was 137/67 mmHg, pulse rate was 97 beats/minute, and body temperature was 36.8°C. His initial hemoglobin level was 15.7 g/dL. Other results of laboratory tests were within normal ranges, including electrolyte level, coagulation test, and cardiac enzyme level. The chest radiograph showed no active lung lesions. We found no evidence of arrhythmic change or ischemic heart disease on the electrocardiogram. The patient underwent upper endoscopy (Figure 1) and we detected diffuse edematous and friable mucosa with deep ulceration and oozing bleeding from the upper (Figure 1(b)) to mid-esophagus (Figures 1(c) and 1(d)). The epiglottis appeared swollen, although he did not complain of dyspnea (Figure 1(a)). Chest CT revealed diffuse mild esophageal wall thickening (Figure 2). Esophageal perforation or mediastinitis was not detected. The patient was diagnosed with acute thermal injury of esophagus by PSMC powder. During his hospital stay, he was treated conservatively, including fasting, empirical antibiotics, and parenteral nutritional support. Seven days after admission, endoscopy revealed that diffuse ulcerative lesions were improved with no more bleeding (Figures 3(a) and 3(b)). He started on a liquid diet and was discharged without any symptoms. Endoscopy one month later showed mild scarring without stricture (Figures 3(c) and 3(d)), and he was free of symptoms.

## 3. Discussion

Acute thermal injuries of the esophagus can be caused by ingestion of hot solid or liquid materials [4–10]. The esophageal thermal injury caused by ingestion of hot liquid materials is characterized by a candy-cane appearance of alternating bands of whitish pseudomembrane and linear erythema [4–6]. On the other hand, esophageal injuries resulting from eating solid foods such as hot jelly roll [7], hamburger [8], and hot steamed eggs [9] appeared as a localized ulcer [7–9]. The reason for the different endoscopic findings from liquid and solid materials is that the candy-cane esophagus is caused by the transient rapid flow of hot liquids, whereas localized ulcers are caused by the pressure effect of hot solids. Therefore, the appearance of thermal injury of the esophagus varies depending on the type of ingested substance. In this case, not only localized deep ulcers but also linear erythema was observed in the esophagus. The reason for these atypical endoscopic findings was that the patient swallowed PSMC powder and drank water, resulting in slow movement of the semiliquid material from the pharynx to the upper esophagus.

**Figure 4:**
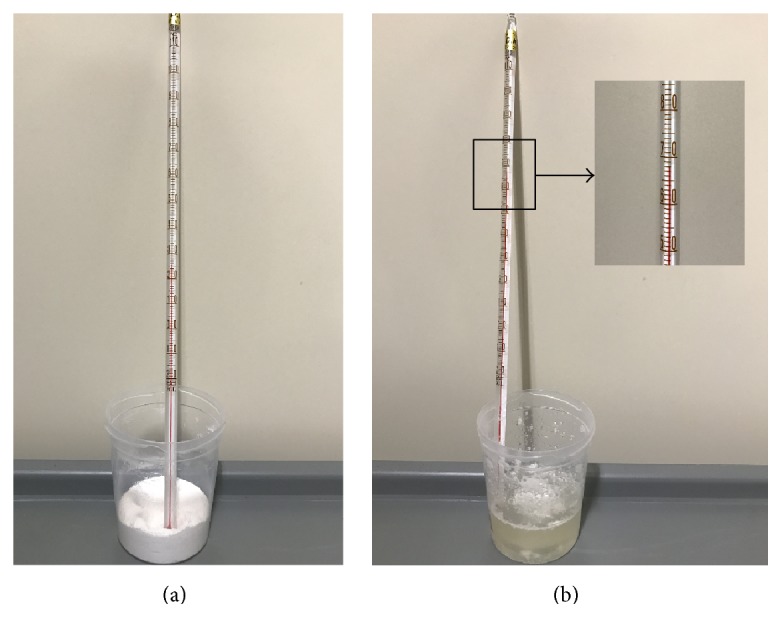
Exothermic reaction of Picolight® powder. (a) A pack of Picolight powder in a plastic container. The temperature of the powder was 25°C. (b) The temperature was measured immediately after mixing the Picolight powder with 30 cc of water. The temperature rose to 68°C.

PSMC is a low volume bowel cleansing agent that was introduced to the Korean market in 2011, although it has been used widely for more than 20 years in European countries [11]. A pack of picosulfate with magnesium citrate (Picolight powder, Pharmbio, Seoul, Korea) consists of 10 mg of sodium picosulfate, 3.5 g of magnesium oxide, and 12 g of anhydrous citric acid. An exothermic reaction occurs when magnesium oxide reacts with anhydrous citric acid to form magnesium citrate (MgO + C_6_H_8_O_7_ + H_2_O → C_6_H_6_MgO_7_ + 2H_2_O). To confirm the exothermic reaction, the authors measured the temperature change after dissolving a pack of PSMC powder in water. When a pack of PSMC powder was poured into 30 mL of water and stirred, it boiled and the temperature rose to 68°C (Figure 4). Third-degree burn of skin can occur when hot water at 69°C contacts the skin for several seconds [12]. Thus, drinking water after swallowing PSMC powder will cause a severe thermal injury to the esophagus. In addition, when PSMC powder is dissolved in a small amount of water, it produces a strong acidic liquid, leading to corrosive esophagitis [13]. Therefore, although PSMC powder is considered safe, incorrect usage may lead to severe side events such as thermal injury of esophagus or stomach. It is recommended that PSMC should be completely dissolved in sufficient quantity of water and cooled before drinking. According to drug instructions, the PSMC powder should be dissolved in 150 mL of water before drinking.

To date, four cases of PSMC-related thermal injuries have been reported in Korea, including our case. The thermal injury occurred in the upper esophagus in our case, and in other cases it occurred in the esophagus and stomach [11, 14] and stomach [15]. Fortunately, all cases recovered with conservative management without complications such as esophageal stricture or perforation. However, considering the fact that PSMC is widely used as a colon cleansing agent and the fact that many cases of thermal injury may not have been reported, clinicians should educate the patients about exactly how to use this medicine to prevent these thermal injuries.

Treatment of esophageal thermal injury is conservative, including fasting, hydration, administration of proton pump inhibitor, and empirical antibiotics. The prognosis is generally favorable [4–10]. No esophageal stricture or perforation has been reported as a complication of esophageal thermal injury.

In conclusion, we report a case of esophageal thermal injury caused by the incorrect use of PSMC. Although acute thermal injury of esophagus has a favorable prognosis, appropriate management and careful observation are needed to prevent further damage. Endoscopists and dispensing pharmacists should educate patients to not swallow PSMC powder for bowel preparation.
